# Circulating miRNAs as diagnostic biomarkers for adolescent idiopathic scoliosis

**DOI:** 10.1038/s41598-018-21146-x

**Published:** 2018-02-08

**Authors:** José Luis García-Giménez, Pedro Antonio Rubio-Belmar, Lorena Peiró-Chova, David Hervás, Daymé González-Rodríguez, José Santiago Ibañez-Cabellos, Paloma Bas-Hermida, Salvador Mena-Mollá, Eva María García-López, Federico V. Pallardó, Teresa Bas

**Affiliations:** 1 Centro de Investigación Biomédica en Red de Enfermedades Raras (CIBERER), Valencia, Spain; 2 Instituto de Investigación Sanitaria INCLIVA, Avenida de Menéndez y Pelayo, 4, 46010 Valencia, Spain; 3 Dept. Physiology. Faculty of Medicine and Dentistry, University of Valencia, Av/Blasco Ibañez, 15, 46010 Valencia, Spain; 4 Instituto de Investigación Sanitaria IISLAFE, Av/Fernando Abril Martorell, 106. Torre A 7, 46026 Valencia, Spain; 5 Unidad de Raquis. Hospital Universitari i Politècnic La Fe, Av/Fernando Abril Martorell, 106, 46026 Valencia, Spain; 6 Unidad de Bioestadística, Instituto de Investigación Sanitaria IISLAFE, Av/Fernando Abril Martorell, 106, 46026 Valencia, Spain

## Abstract

The aetiology of adolescent idiopathic scoliosis (AIS) has been linked to many factors, such as asymmetric growth, neuromuscular condition, bone strength and genetic background. Recently, epigenetic factors have been proposed as contributors of AIS physiopathology, but information about the molecular mechanisms and pathways involved is scarce. Regarding epigenetic factors, microRNAs (miRNAs) are molecules that contribute to gene expression modulation by regulating important cellular pathways. We herein used Next-Generation Sequencing to discover a series of circulating miRNAs detected in the blood samples of AIS patients, which yielded a unique miRNA biomarker signature that diagnoses AIS with high sensitivity and specificity. We propose that these miRNAs participate in the epigenetic control of signalling pathways by regulating osteoblast and osteoclast differentiation, thus modulating the genetic background of AIS patients. Our study yielded two relevant results: 1) evidence for the deregulated miRNAs that participate in osteoblast/osteoclast differentiation mechanisms in AIS; 2) this miRNA-signature can be potentially used as a clinical tool for molecular AIS diagnosis. Using miRNAs as biomarkers for AIS diagnostics is especially relevant since miRNAs can serve for early diagnoses and for evaluating the positive effects of applied therapies to therefore reduce the need of high-risk surgical interventions.

## Introduction

Adolescent idiopathic scoliosis (AIS, OMIM #181800) is the commonest spinal abnormality in children, and is a three-dimensional spine deformity that causes a coronal imbalance of 10 degrees and above. Indeed 70–80% of diagnosed scoliosis cases in adolescents are idiopathic. Clinically, AIS is characterised by aesthetic deformity and alterations in pulmonary function, sometimes with pain and psychological effects. A recent epidemiology report has demonstrated a significant AIS burden in the USA and Europe that affects 2–3% of the population aged under age 18, with more than 1 million cases in Europe^1^ and about 7 million in the USA, and an associated hospital cost of US$ 2.7 billion^2^.

The aetiology of AIS is currently unknown. The role of the genetic factors involved in AIS is widely accepted^3^, and AIS has been suggested to be possibly caused by the effect of multiple genes in various ethnic populations^4–6^. However, the implication of epigenetics in the aetiology of AIS has been recently proposed^7^.

Epigenetics is defined as changes in gene activity and expression that occur with no alterations in DNA sequences, caused by DNA methylation, histone post-translational modifications, and non-coding RNAs (i.e., miRNAs). The environment, nutrition and lifestyle are some factors that can modulate the epigenome, and can thus contribute to AIS progression. In line with this, effects of the so-called exposome (how the environment interacts with our epigenome) may contribute to a proper formed and balanced spine, which may also lead to altered spinal growth and formation that cause AIS^7^.

Epigenetics has had a huge impact on biomedical research and is providing new biomarkers for the diagnosis and prognosis of diseases. It is also contributing to analyse the molecular causes that underlie diseases. In this context, miRNAs are very promising biomolecules to be used as biomarkers^8,9^ because miRNAs act as signalling molecules and participate in many biological processes, such as cellular development, differentiation and apoptosis. The good stability of circulating miRNAs in the RNase-rich environment of the bloodstream^10,11^, and also in different biospecimens used in clinical routine^12^, make these biomolecules an optimal source of candidate biomarkers. In fact miRNAs have been demonstrated as valuable biomarkers in a wide variety of human diseases^13^.

miRNAs are a large family of short non-coding RNAs (17–25 nucleotides) which participate in gene regulation through their binding capacity to mRNA. miRNAs have been proposed as important contributors in bone morphogenesis and osteoclastogenesis (OsteomiRs)^14^, which make them interesting biomolecules to study the molecular causes of AIS. In the present-day no clear explanation for the origin of AIS is available, and genetic analyses do not completely explain the existing variability between patients. Thus epigenetics can help to improve the characterisation of these patients by providing new biomarkers of AIS.

The diagnosis and prognosis of AIS is currently a challenge. AIS is a condition with an unpredictable and difficult natural history, and it is challenging to know which patients will require conservative or surgical treatment^15^.

Previous studies have attempted to use genetic polymorphisms related to AIS as biomarkers for early diagnosis and prognosis. Moreover, genes such as fibrillin-1 (FBN1) and fibrillin-2 (FBN2) have been found to be associated with severe AIS^16^. A genome-wide association study has identified the ladybird homeobox 1 (LBX1) locus as being associated with AIS susceptibility in both Asian and non-Hispanic white populations^5,17^. Polymorphisms in the transforming growth factor beta (TGF-β) gene have also been proposed to be associated with AIS susceptibility and curve severity in a Russian population^18^. Recently, a new genome-wide association study in a Chinese population identified a novel susceptibility loci, which highlighted the importance of the Wnt/beta-catenin pathway in AIS^19^.

Regarding epigenetic mechanisms, Liu *et al*., have recently identified 139 lncRNAs (long non-coding RNAs) in blood, which were differentially expressed in the AIS patients compared with the control individuals^20^. More recently, Sun *et al*., proposed using the lipid metabolic profile for diagnosing AIS^21^.

Many treatments consist in exercise, braces and surgical interventions of the spine, which are invasive and costly. It is difficult to predict the clinical evolution of AIS by X-ray examinations with patients (the current gold standard), and it is even more difficult to decide the best time for initial treatment with braces or surgery. Furthermore, patients who suffer from scoliosis are exposed to an average of 23 radiographs during a 3-year period^22^. Ronckers *et al*. followed 5,513 females, who were exposed to an average of 23 radiographs during the treatment and follow-up of scoliosis, and found that the risk of mortality was 46% higher in the AIS patients than in the general population, where cancer was the primary cause of death in about 23% of cases^23^. In a more recent study, Simony *et al*. found that the overall cancer rate in an AIS cohort treated 25 years earlier was 4.3%, which is 5-fold higher compared to an age-matched Danish population, and endometrial and breast cancers were the most frequent^24^. Along these lines, increasing knowledge on the physio-pathological events that take place in AIS and about the discovery of molecular biomarkers is necessary to generate new accurate and risk-free diagnostic tools^25^.

Here we provide evidence for the epigenetic deregulation in AIS of a series of miRNAs detected by Next Generation Sequencing (NGS) and validated by RT-qPCR. The employed computational methods determine that those miRNAs detected by NGS participate in bone metabolism and, therefore, link epigenetics with the aetiology of AIS. Finally, a blood circulating 4-miRNA signature with good sensitivity and specificity is proposed for a molecular-based AIS diagnosis.

## Results

### Clinical description of AIS patients

Thirty patients and 13 age-matched healthy subjects were included in the present research study. The mean age of the patient group was 15.02 years old (±2.06). The female-male ratio was respectively 5:1. The mean BMI value of this group was 19.84 (±3.03). Familiar history was positive for 13 patients, which represents 43.33% of the whole group (Table 1). The mean age at diagnosis was 10.65 years old (±1.78). As previously mentioned, the minimum follow-up was 2 years. The mean age at menarche was 12.26 years old (±1.82). At the time blood tests were done, two patients still presented no menarche. The Risser evaluation for patients was 2.46 (±1.80). Further additional clinical and radiological features of the AIS patients are provided in the Supplementary Information. According to the Cobb angle, the distribution of all the patients was seven patients with mild scoliosis (23.3%), 17 with moderate scoliosis (56.7%) and six with severe scoliosis (20.0%).

**Table 1:** Comparison of the mean values for age, BMI (body mass index), Risser, SF-36 questionnaire and family history percentage between both study groups used for miRNA discovery. SD, standard deviation.

	AIS PATIENTS *Mean* ± *SD*	CONTROL GROUP *Mean* ± *SD*
AGE	15.02 ± 2.06	13.69 ± 1.97
FEMALE-MALE (RATIO)	5:1	1.17:1
BMI	19.84 ± 3.03	20.4 ± 2.51
FAMILY HISTORY	43.33%	38.46%
RISSER	3.46 ± 1.10	2.46 ± 1.80
COBB ANGLE	32.93° ± 19.38	2.23° ± 2.68
SF-36	83.56 ± 14.07	85.93 ± 8.04

Thirteen healthy individuals composed the control group, in which no scoliosis sign was detected during the physical examination procedures. The mean age of this group was 13.69 years old (±1.97). Unlike the patients group, the female-male ratio was more balanced: 1.17:1 respectively. Their mean BMI was 20.4 (±2.51). Five individuals had a positive familiar history of idiopathic scoliosis, which meant 38.46%. Of the six females, three still presented no menarche.

For validation purposes, we enrolled a new independent cohort which comprised 17 patients (4 males and 13 females; a ratio of 1:3) and seven controls (2 males and 5 females; a ratio of 1:2.5), following the prevalence data described by Konieczny *et al*.^1^.

The mean age of the patients group was 14.65 years old (±2.66). The mean BMI value for this group was 20.35 (±3.02). The Risser evaluation was 3.83 (±1.90). According to the Cobb angle, the distribution of all the patients was seven with mild scoliosis (41.2%), five with moderate scoliosis (29.4%) and five with severe scoliosis (29.4%). For the control group of the validation cohort, the mean age was 14.67 years old (±1.54), and scoliosis was not referred to.

### Identification of differentially expressed miRNAs using Next Generation Sequencing

To detect the potential AIS biomarkers, we examined the NGS data obtained after sequencing the miRNAs isolated from the plasma of the screening cohort, which was composed of AIS patients (n = 17) and healthy subjects (n = 10).

**Figure 1:**
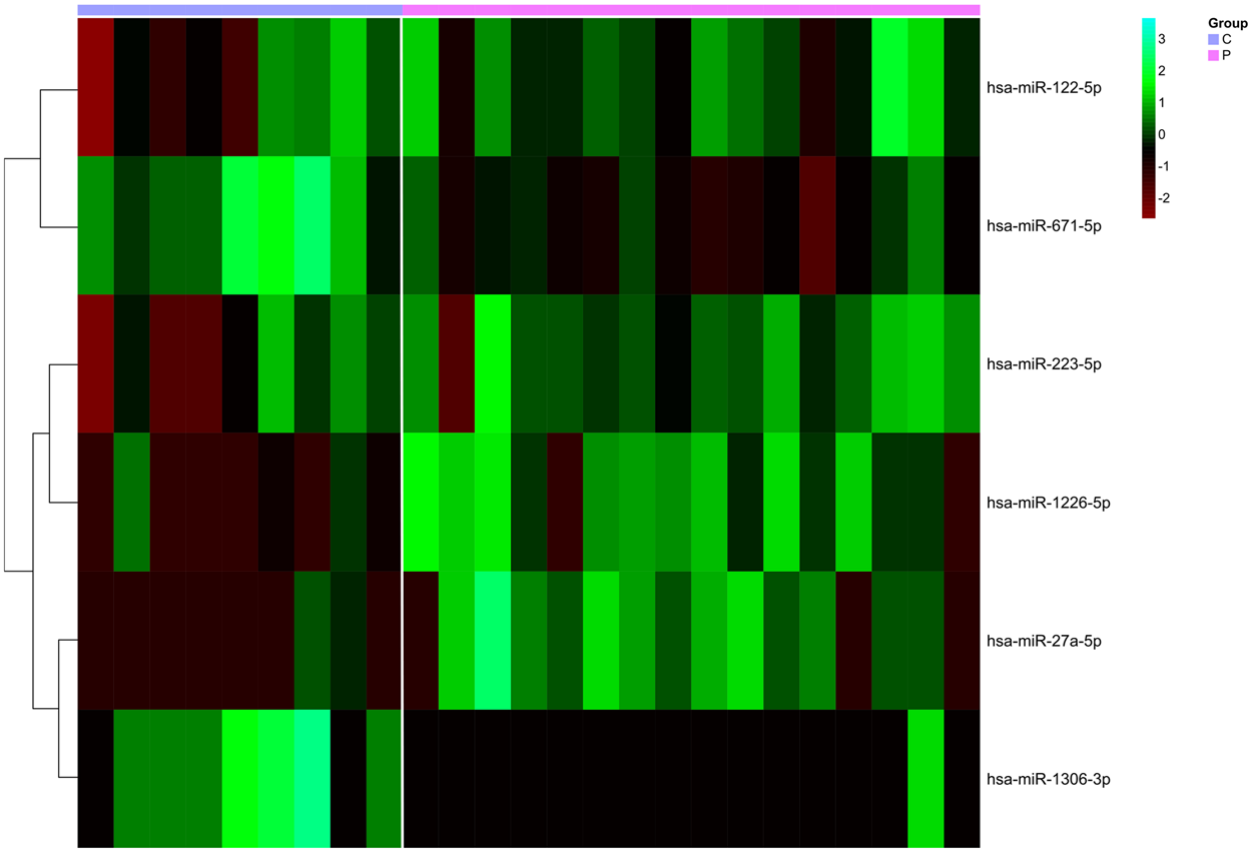
Heatmap with the hierarchical clustering of differentially expressed miRNAs in AIS. Expression levels of the miRNAs selected by the random forest analysis and the Robinson and Smyth test. Raw count values were log-transformed and samples were ordered according to their corresponding group: Controls (C) or AIS patients (P).

Our random forest model results showed that the circulating miRNAs from the AIS patients presented differential expression patterns compared to the controls. A signature formed by six miRNAs was able to distinguish patients from controls, which provided molecular information about the role of these miRNAs in this pathology. Our random forest model achieved a cross-validated accuracy of 100% (100% sensitivity and 100% specificity). It selected miR-122-5p, miR-671-5p, miR-223-5p, miR-1226-5p, miR-27a-5p and miR-1306-3p as the most important predictors of the disease. The results of our model are depicted on a heatmap (Fig. 1). miR-671-5p and miR-1306-3p were underrepresented, while miR-1226-5p and miR-27a-5p were present at high levels in the plasma of the patients compared to the controls. miR-223-5p and miR-122-5p were homogenously overrepresented in the patients, but their expression was heterogeneous among the controls. The Robinson and Smyth exact negative binomial test results reinforced our random forest model outcomes since three of the previously selected miRNAs also showed a statistically significant differential expression among the groups: miR-122-5p, miR-671-5p and miR-223-5p.

### Validation of the differentially expressed miRNAs by RT-qPCR

To validate the miRNAs which were differentially expressed in the patients compared to the controls, we conducted RT-qPCR using specific TaqMan Small RNA assays (Table 2), which included not only additional AIS patients, but also additional healthy subjects. We analysed these miRNAs in 30 patients and 17 healthy adolescent subjects (validation cohort).

**Table 2:** miRNAs selected as biomarkers for AIS.

miRNA name	Mature sequence	Accession	TaqMan Small RNA Assays
hsa-miR-671–5p	aggaagcccuggaggggcuggag	MIMAT0003880	197646_mat
hsa-miR-1306–3p	acguuggcucugguggug	MIMAT0005950	241056_mat
hsa-miR-1226–5p	gugagggcaugcaggccuggaugggg	MIMAT0005576	002758
hsa-miR-27a-5p	agggcuuagcugcuugugagca	MIMAT0004501	002445
hsa-miR-223–5p	cguguauuugacaagcugaguu	MIMAT0004570	002098
hsa-miR-122–5p	uggagugugacaaugguguuug	MIMAT0000421	002245
hsa-miR-191–5p	caacggaaucccaaaagcagcug	MIMAT0000440	002299

**Figure 2:**
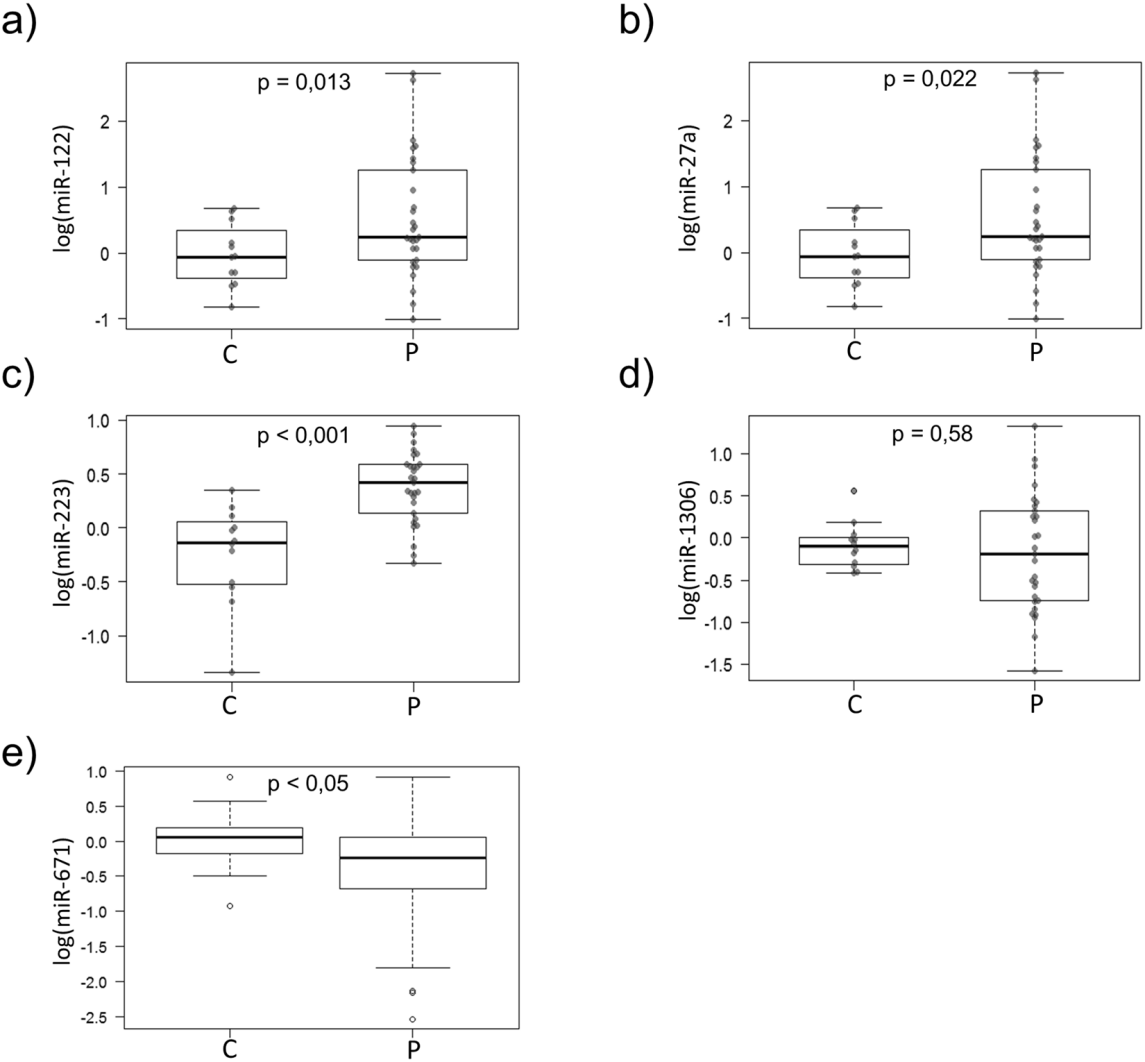
Relative expression levels of the miRNAs with different representations found in the plasma of the AIS patients *versus* the control healthy subjects. Box plot of the relative expression levels of the miRNAs analysed by RT-qPCR, normalised to miR-191 as an endogenous control and calculated using the 2^-∆∆Ct^ method. (**a**) miR-122 (Fold Change, FC > 2.65; p < 0.05; (**b**) miR-27a (FC > 1.87; p < 0.005); (**c**) miR-223 (FC > 1.5; p < 0.0001); (**d**) miR-1306 (FC > 1.03; p = 0.38); and (**e**) miR-671 (FC > 0.81; p = 0.53). Samples were ordered according to their corresponding group: controls (C) or AIS patients (P). An independent samples t-test was applied to analyse the biospecimens from the 17 healthy subjects (6 males and 11 females) and the 29 AIS patients (5 males and 24 females). A p < 0.05 was considered to indicate a significant difference.

The log_2_ was calculated to compare the relative expression level of each miRNA in both the patients and controls. hsa-miR-191 was used in this study as a reference because we observed similar read counts among all the NGS-analysed samples. We were able to detect all the miRNAs (Ct < 35) that derived from the previous NGS study, except for miR-1226-5p. According to the NGS results, three of the miRNAs (miR-122-5p, miR-27a-5p, and miR-223-5p) showed higher levels in the patients than in the healthy subjects (Fig. 2). miR-671-5p and miR-1306-3p did not show any significant differences between the patients and the controls.

We also performed a study with an independent cohort of 17 AIS patients (4 males and 13 females; a ratio of 1:3) and seven healthy adolescents as the controls (2 males and 5 females; a ratio of 1:2.5). We carried out a qRT-PCR analysis of the miRNA signature and found that miR-27a-5p, miR-223-5p, miR-671-5p, miR-1306-3p showed higher levels in the patients than in the healthy subjects. (Supplementary Figure S1). miR-122-5p showed a tendency to be overexpressed, but it was not statistically significant up-regulated in the patients in the independent cohort.

### Assessing the diagnostic potential of the validated miRNA

**Figure 3:**
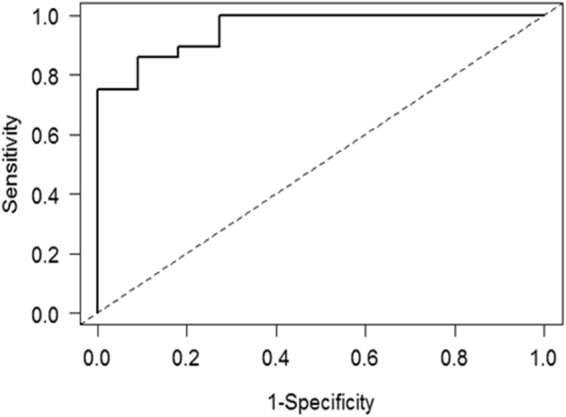
Receiver operating characteristic curve analysis of the 4-miRNA signature validated by RT-qPCR for diagnosing AIS. The model uses a panel of 4-miRNA signature composed of miR-122, miR-27a, miR-223 and miR-1306, and achieved an AUC value of 0.95 (CI: 0.89-1). At a fixed specificity of 90%, the model achieved a sensitivity of 85.7%.

To assess the potential use of the miRNAs as diagnostic biomarkers of AIS, the receiver operating characteristic curve (ROC) was performed on the validation data obtained by RT-qPCR. Our model used a 4-miRNA signature composed of miR-122-5p, miR-27a-5p, miR-223-5p and miR-1306-3p, and achieved an area under the curve (AUC) value of 0.95 (CI: 0.89-1), a high sensitivity of 92.9% and specificity of 72.7% (Fig. 3). The probability of a specific patient having AIS can be predicted by our signature with the following equation (1):

Algorithm to calculate the probability of suffering Idiopathic Scoliosis (AIS) in a human subject according to the determination of circulating miRNA levels determined by qRT-PCR.

1 $$\Pr (AIS)=\frac{{e}^{-7.11+1.50\ast miR122+1.15\ast miR27a+6.30\ast miR223-3.08\ast miR1306}}{1+{e}^{-7.11+1.50\ast miR122+1.15\ast miR27a+6.30\ast miR223-3.08\ast miR1306}}$$

### Analysis of the miRNAs targets and pathway study in the bone metabolism context

Firstly, we analysed the targets of these miRNAs using miRTarBase, v7.0, which collects experimentally validated miRNA-target interactions^26^. However, as the number of the experimentally validated miRNA targets was limited, we used different and computational methods to predict miRNA targets^27^, such as miRanda, DIANA microT-CDS and TargetScan, v6.2. In order to obtain the list of Entrez genes predicted to be targeted by miRNAs, we identified the differentially expressed genes and validated them by RT-qPCR.

**Figure 4:**
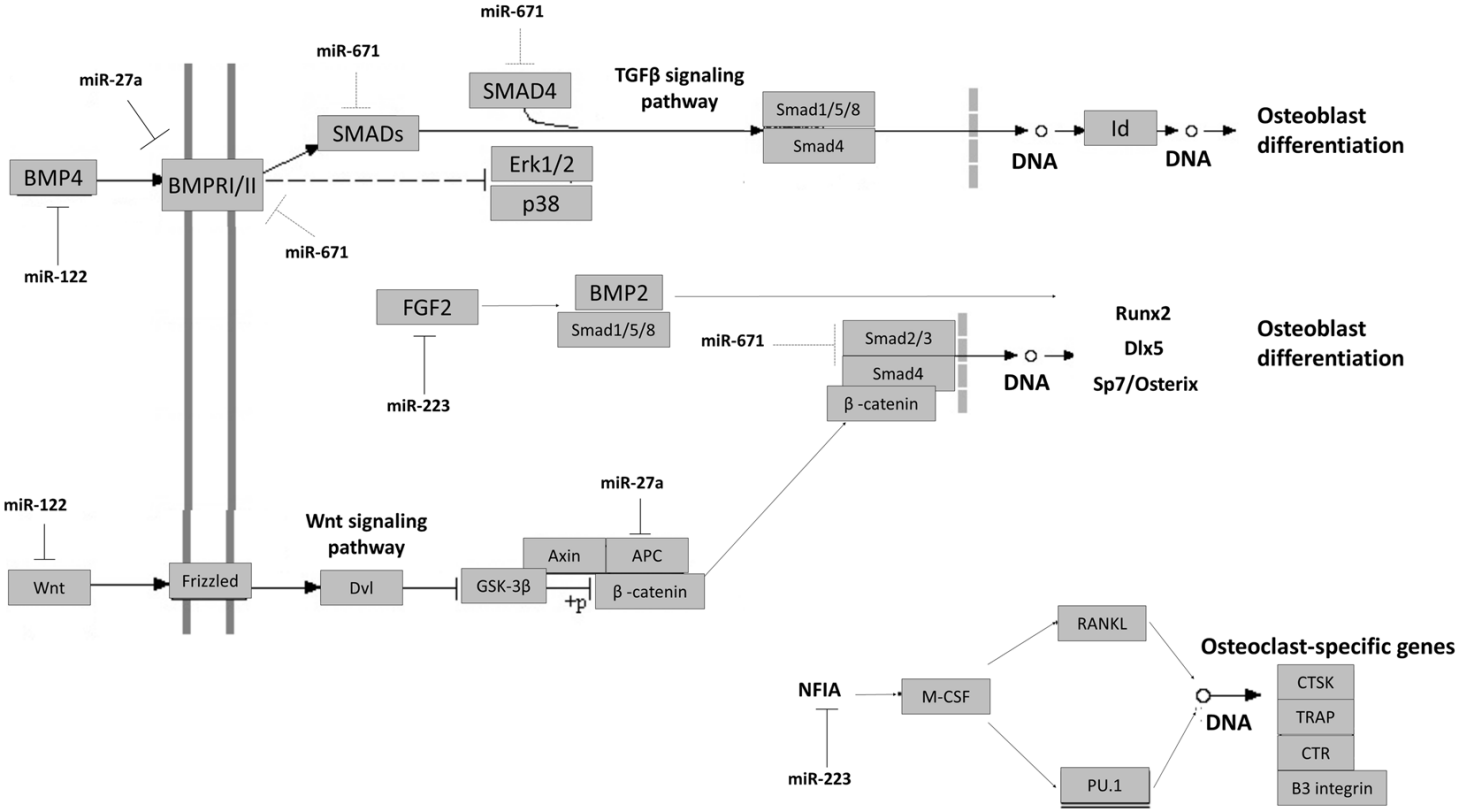
Schematic representation of the gene targets of miRNA identified by NGS in the AIS patients and their role in osteoblast and osteoclast differentiation depicted from KEGG and DIANA miRPath v3. The continuous blunt end line indicates the gene interactions with the overexpressed miRNA. The dotted grey blunt end line indicates the gene interactions with the down-regulated miRNA. Osteoblast progenitor formation is mediated by Smad cascade signalling through the activation of the TGFβ and Wnt signalling pathways. Smad become phosphorylated and migrates into the nucleus, where it induces Runx2, Dlx5 and Sp7/Osterix transcription to induce the differentiation of osteoblasts. In these intricate mechanisms, microRNAs can modulate key genes to control osteoblast and osteoclast differentiation. miR-671–5p blocks BMPR2 and Smad2/3, which are key regulators on this pathway. So the down-regulation of this miR-671 may contribute to the osteoblast differentiation pathway. mir-27a also blocks BMPRI/II and thus produces the inhibition of this pathway, which is also affected by the miR-122 interaction with BMP4. However, the dual role of miR-27a can activate osteoblast differentiation by the activation of Wnt signalling through the inhibition of APC. In such circumstances APC do not form a complex with glycogen synthase kinase 3 (GSK3B), so the levels of non-phosphorylated β-catenin increase and migrate to the nucleus, where it associates with members of the TCF/LEF transcription factors by controlling the gene transcription of RUNX2. However, osteoblast differentiation may undergo the upstream regulation of Wnt by miR-122, which alters this pathway. Finally, miR-223 may contribute to affect osteoclast -specific genes transcription and the maturation of functional osteoclasts by inhibiting NFIA, the inhibitor of osteoclast differentiation, which results in increased osteoclast activation.

We also analysed biochemical networks, which were regulated by our validated miRNAs. We used the DIANA-miRPath v3.0 software, and the Kyoto Encyclopedia of Genes and Genomes (KEGG) pathway analysis, to find the significantly enriched targets of the selected miRNAs. In this way we identified molecular signalling pathways by regulating the pluripotency of stem cells (hsa04550) with a p-value of 1.08e^−5^. On this pathway, five of our miRNAs participated by regulating up to 40 genes. According to the interactions between miRNAs and the target genes, we built a miRNA-gene-network which illustrates the key regulated pathways (Fig. 4). We centred our search on bone morphogenic protein (BMP) and Wnt pathways (hsa04310), which are extremely important in osteoblast/osteoclast differentiation and bone metabolism.

We particularly found that miR-122-5p counteracts with Wnt1 to suppress the activation of the Wnt/β-catenin pathway, as previously demonstrated by Wang *et al*.^28^. The Wnt/β-catenin pathway is the canonical pathway in osteoblast differentiation, on which the activation of β-catenin promotes osteoblast commitment from mesenchymal progenitors by stimulating osteoblast proliferation and differentiation, and preventing osteoclastogenesis, as well as osteoblast and osteocyte apoptosis^29^. The tumour suppressor adenomatous polyposis coli protein (APC) participates in Wnt signalling, which brings β-catenin to glycogen synthase kinase 3B (GSK3B) to result in the phosphorylation of β-catenin and its degradation by the proteasome. The phosphorylation of β-catenin impedes its migration to the nucleus, and leads to reduced bone formation and increased bone resorption^30^. It has been described that miR-27a inhibits the gene expression of the APC protein by enabling β-catenin migration to the nucleus, and thereby activating osteoblast differentiation. The expression levels of miR-27a also correlate positively with β-catenin^31^. The relevance of hsa-miR-27a was quite clear as TargetScan Human 6.2 predicted 21 targets that contained 23 conserved binding sites for the genes on the Wnt/β-catenin pathway. Osteoblast differentiation is also promoted thanks to the participation of bone morphogenic proteins, such as BMP-2, BMP-4^32,33^, and in a modest manner by BMP-8^33^, and their receptors BMPR1A and BMPR2. DIANA-miRPath v3.0 have shown that BMP and BMPR1a are targets of miR-27a-5p. Hence this miRNA dramatically inhibits osteogenesis by the repression of BMP and SMAD signalling^34^ (Fig. 4).

The DIANA-miRPath software, v3.0, also showed the interaction of miR-223-5p with the FGF2 (fibroblast growth factor 2) gene, which positively modulates osteoblast differentiation and bone formation through the Wnt/β-catenin pathway^35^, which is necessary for BMP2-induced nuclear accumulation and the co-localisation of phospho-Smads1/5/8 and Runt-related transcription factor 2 (Runx2) transducers during bone formation^36^ (Fig. 4). miR-223-5p targets PCGF3, also known as BMI1, a key gene involved in the self-renewal of bone marrow mesenchymal stem cells, and plays a critical role in promoting osteogenesis. In fact neonatal BMI1 knock-out mice exhibit skeletal growth retardation, reduced chondrocyte proliferation and increased apoptosis^37^. miR-223-5p can regulate osteoclastogenesis throughout the down-regulation of osteoclast marker genes PU.1, RANKL, NFATc1, TRAP, c-Jun, cathepsin K^38^.

Our pathway analysis also detected the interaction of miR-671-5p with SMAD3 and SMAD4, which participate in the Smad-dependent transcription of Runx2 that is critical for osteoblast specific gene transcription (e.g. Runx2, Dlx5 and Sp7/Osterix) and for skeleton formation^39^. We also looked for other interactions of miR-671-5p using TargetScan, and we predicted the interaction of miR-671-5p with both BMPR2 and bone morphogenic BMP8A and BMP8B transcripts. In this way, the interaction of miR-671-5p with BMPR2 has been recently described^40^. Furthermore, as predicted by DIANA-microT-CDS, miR-671-5p targets Smad3, which is a critical component of the TGFβ pathway that plays a key role in bone formation. In fact Smad3 overexpression enhanced the levels of Runx2, also known as core-binding factor subunit alpha-1 (CBFα-1), Sp7/Osterix and β-catenin, which are the master transcription factors for osteocytic differentiation (Fig. 4). They regulate the differentiation of either mesenchymal cells or bone marrow stromal cells to osteoblasts^41^.^42^^,^

Finally, by filtering the results using DIANA-miRPath, v3.0, we found that miR-1306-3p targeted the protein-serine/threonine phosphatase 2CB (PP2CB), which participate on the TGFβ signalling pathway (hsa04350). miR-1306 also targets Rac2, as shown on the Wnt signalling pathway (hsa04310), which is an essential Rho GTPase in mature osteoclasts for chemotaxis and resorptive activity^43^. The target prediction for miR-1306-5p, for which DIANA-microT-CDS was employed, found that it targeted bone morphogenic protein 1 (BMP1), which is involved in bone and cartilage development^44^.

## Discussion

Early AIS diagnosis will potentially improve treatments and reduce the number and risk of surgical intervention. However, accurate diagnostics and prognostics using real-time biomarkers is currently a challenge^21^.

The role of miRNAs is attracting much interest in bone metabolism and osteoblast/osteoclast differentiation as it miRNAs have been described to be involved in bone mass disorders^14^.^45^^,^ AIS patients are characterised by low bone mineral density and abnormal skeletal growth^46^. Therefore, it is very important to maintain the optimal molecular control of bone formation and ossification during spine maturation. In physiological terms, osteocytes are crucial for the spine’s mechano-construction/mechano-stress response. In addition, osteoblasts lead to the inhibition of osteoclastic bone resorption, mineralisation and the mechanical strengthening of bone^47–49^, which spells growing concern about the importance of the correct balance of osteoblastic/osteoclastic signals during spine maturation. Abnormal skeletal maturation has also been observed in AIS patients^46,50^. Epigenetics has been proposed to be implicated in the aetiology of AIS, which suggests that environmental factors seriously contribute to AIS progression^7,51^ miRNAs are involved in many biological processes that can epigenetically regulate cellular development and differentiation. Several studies have shown that miRNAs regulate osteoblastic differentiation^52,53^, maturation and/or osteocytic differentiation^54^. Ell *et al*. have described a number of miRNAs as regulators of bone homeostasis and bone metastasis^55^, and a key role of miRNAs in osteogenic differentiation for healthy and pathological conditions has been described^56^.

Our small-RNA sequencing studies found that a series of circulating miRNAs were differentially represented in the AIS patients compared to the healthy subjects, which were validated by RT-qPCR in two independent cohorts (miR-122-5p, miR-27a-5p, miR-223-5p, miR-1306-3p, and miR-671-5p). With a 4-miRNA signature composed of miR-122-5p, miR-27a-5p, miR-223-5p, and miR-1306-3p and the validation cohort, we demonstrated a high sensitivity of 92.9% and a specificity of 72.7% with an AUC value of 0.95 (CI: 0.89-1), which is higher than those obtained by standard methods; i.e. the Adam’s test^57^. The classical method also relies on the observer’s experience, and on the type of spinal curves and Cobb angles measured from different radiographs. Although X-rays are currently considered the gold standard for AIS examinations, scoliosis screening using chest radiographs involves limited values of sensitivity and specificity^58^, and many health risks (i.e., breast cancer^23^ and breast and endometrial cancer^24^).

It is noteworthy that the miRNAs which we discovered actually targeted the genes that participate in the cell signalling pathways that regulate the pluripotency of stem cells (KEGG: hsa04550). In this context, miR-122-5p targets the Wnt1 suppressing activation of the Wnt/β-catenin pathway^28^. The experimental data reported by Mizuno *et al*., have shown that miR-122 can down-regulate BMP4^59^. Therefore, this pathway also inhibits the activation of osteoblast differentiation. However, miR-122-5p is also involved in the mineralisation of smooth muscle cells (SMC), which play an integral role in skeletal bone formation by undergoing trans-differentiation to osteoblast-like cells and by diminishing the activity of osteoclast-like cells^60^. This ambiguous role associated with miR-122 is compensated by the effect of miR-27a, since it activates osteogenesis through the activation of the Wnt signalling pathway by targeting APC and allowing β-catenin migration to the nucleus. This contributes to the differentiation of mesenchymal stem cells into osteoblasts^31^ and, in turn, increases bone mass^30^. Our results agree with the findings of Zhu *et al*., which highlight the relevance of the Wnt/beta-catenin pathway in AIS development^19^, which reinforces the role of this pathway in AIS. In fact LBX1 and PAX3, which are both involved in the downstream of the Wnt/beta-catenin pathway, have been reported as susceptible genes of AIS^61,62^. It has also been established that miR-27a inhibits adipocyte formation when overexpressed by blocking the transcription of two main regulators of adipogenesis, the peroxisome proliferator-activated receptor gamma (PPARγ) and C/EBPα^63^. Hence when miR-27a inhibits adipogenesis, it activates osteogenesis through the above-described pathway. As shown in the previous section, miR-223-5p targets FGF2 and PCGF3 or BMI1, and thus interferes with osteoblast differentiation^35^.^37^^,^ Furthermore, miR-223–5p regulates osteoclastogenesis in RAW 264.7 cells^38^. Using a mouse osteoclast precursor (RAW264.7 cells), Sugatani and Hruska found that miR-223 overexpression produced the down-regulation of osteoclast marker genes; PU.1, RANKL, NFATc1, TRAP, c-Jun, cathepsin K;^38^ by blocking osteoclastogenesis, osteoclast development and bone destruction^64^. Interestingly, miR-223-5p can be modulated by C/EBPα^65^, which is controlled by miR-27a. Contradictorily, miR-223 has been shown to down-regulate NFIA, an inhibitor of osteoclast differentiation, which results in an increased M-CSF function that produces the activation of osteoclastogenesis factors RANKL and PU.1^14^. As previously mentioned however, the up-regulation of miR-27a may prevent C/EBPα action, and could thus equilibrate osteoclastogenesis inhibition. Therefore, miR-223 may contribute synergistically to osteoclast development and bone destruction^38^. Contrarily to the down-regulation of miR-671 in the first analysed cohort, we found that miR-671 was up-regulated in the second independent cohort. This miRNA targets transcripts BMPR2 and BMP8A and BMP8B, which are bone morphogenic proteins, by participating in endochondral osteogenesis *in vivo*, which hence indicates bone inductive activity^66^. miR-671-5p also targets Smad3, a critical component of TGFβ. So Smad3 overexpression enhances the levels of RUNX2 and other transcription factors required for the differentiation of either mesenchymal cells or bone marrow stromal cells to osteoblasts^41,42^^,^.

All in all, our results indicated an imbalance in miRNA-mediated signalling during osteoblast/osteoclast differentiation in AIS patients, which thus alters the correct homeostasis of bone destruction and bone formation.

As far as we know, this is the first report of the comprehensive interrogation of circulating miRNAs as biomarkers in AIS patients. Our results demonstrated that circulating miRNAs in plasma can be potentially used as biomarkers for AIS, which provides a new method for diagnosing AIS that would at least reduce repetitive X-ray irradiation for disease progression monitoring. We found that six miRNAs, which are closely related with bone metabolism, were differentially expressed between the AIS patients and the control subjects. Five of those miRNA were validated by RT-qPCR and checked for implementing a new algorithm to predict AIS. Finally with a selected 4-miRNA signature based on the use of miR-122a-5p, miR-27a-5p, miR-223-5p, and miR-1306-3p, we were able to discriminate the AIS patients from the healthy subjects with high sensitivity and specificity. In conclusion our study yielded two relevant results: 1) evidence for alterations in osteoblast/osteoclast metabolism in AIS mediated by pathogenic miRNAs; 2) we propose an X-ray-free method based on an epigenetic blood circulating 4-miRNA signature for biomolecular-based AIS diagnosis.

## Methods

### Study design and population

This work is a prospective study based on an experimental analysis of the epigenetic profile of AIS. The inclusion criteria for the patients group were: diagnosed for AIS with a Cobb angle > 10° and marked scoliosis; minimum 2-year follow-up; no previous surgical treatment; available radiographies; aged between 12–18 years. The exclusion criteria were: smoker; active infectious or inflammatory process during sample extraction; antioxidants intake; neurologic pathology; congenital syndrome pathology; patients with scoliosis due to secondary causes.

The patients with AIS and the healthy subjects were enrolled in our study after Biomedical Research Ethics Committee (CEIB) of the University and Polytechnic Hospital La Fe approval (registry number 2012/0392 and its revision and modification in 11/21/2016). The informed consent to create a public sample repository of AIS in the CIBERER Biobank (www.ciberer-biobank.es) was also obtained from each patient (Spanish Biobank Registry number: B.0000590). Additional samples from the healthy subjects were obtained from the IBSP-CV Biobank (RVB16024SI) after obtaining informed consent from each participant and the Ethics Committee’s approval of the Comité Ético de Investigación Clínica de la DGSP y CSISP.

All the participants in this study gave their written consent to use their samples and associated information for research purposes, following the standards set by the CIBERER Biobank. All the experimental procedures, protocols and methods were performed in accordance with relevant clinical guidelines and regulations, following standard operation procedures, and with the approval of the Ethics and Scientific Committees.

### Physical and radiological examinations

Physical examination consisted in measuring the following parameters: age, gender, and body mass index (BMI) (kg/cm^2^) (Table 1). A complete neurological examination, including motor and sensory balance, abdominal reflexes, as well as patellar and Achilles reflexes, was performed.

Clinical coronal and sagittal balance was evaluated by the Plumb test. Vertebral rotation assessment on Adam’s Test was evaluated using the Scoliosis Research Society (SRS) scoliometer. Finally, spine deformity was measured by the trunk aesthetic clinical evaluation (TRACE) form, which consists in shoulder, scapular, thoracic and pelvic asymmetry assessments. The physical evaluation for the control group was the same, but excluded the TRACE form.

A radiological study was done for all the included patients and controls, based on two standing X-rays, anteroposterior and lateral views (details of the radiological study are described in the Supplementary Information). According to the SRS criteria, for the present study, a scoliosis diagnosis was considered when the coronal value of deformity was above 10 Cobb degrees. No X-rays images were taken for the healthy subjects group. An independent cohort of healthy subjects was obtained from the BSP-CV Biobank.

Finally, all the included individuals had completed the scoliosis and general health questionnaires, specifically SRS-22, CAVIDRA and SF-36 for the patients group, and SF-36 for the control group.

### Circulating miRNA profiles analysis

Since there is no clear explanation for the origin of AIS, and as a simple genetic analysis does not fully explain the physiopathology and prognosis of AIS, we evaluated epigenetic regulators by NGS.

We isolated cell-free total RNA (including miRNAs) and analysed miRNAs from the AIS patients (n = 17) and healthy subjects (n = 10) by NGS. We also analysed the miRNAs levels using a validation cohort (n = 30) and healthy subjects (n = 13) by RT-qPCR.

#### RNA extraction and quantification

Blood samples were collected from both the AIS patients and healthy subjects in EDTA tubes. Each sample was centrifuged at 2,500 rpm for 10 minutes to separate the plasma and then stored at −80 °C until RNA extraction. We isolated cell-free total RNA (including miRNAs) from 500 uL of plasma with the miRNeasy Serum/Plasma kit (Qiagen, CA. USA), according to the manufacturer’s protocol with some modifications. RNA was eluted with 25 µL of RNAse-free water. The concentration of the cell-free total RNA (including miRNAs) was quantified in a NanoDrop ND 2000 UV-spectrophotometer (Thermo Scientific, Wilmington, DE, USA).

#### Library Preparation and Next-Generation Sequencing

cDNA libraries were constructed using the Ion Total RNA-Seq Kit v2 from Life Technologies and according to the manufacturer’s recommended protocol. The cell-free RNA from the plasma miRNA samples was run in a microfluidics-based platform Agilent 2100 Bioanalyzer to assess the yield and size distribution of RNAs by the Agilent Small RNA Assay. Next 15 ng of miRNA was hybridised with Ion Adapters. Hybridised samples were then mixed with a reverse transcriptase master mix to generate cDNA libraries. The purified cDNA libraries were then amplified by PCR using Platinum PCR Super-Mix High Fidelity and Ion Xpress Barcode reverse and forward primers. The amplified cDNA libraries were purified using nucleic acid binding beads, binding buffers, and were run in an Agilent 2100 Bioanalyzer to determine the yield and size distribution of each library.

Approximately 10 pM of the pooled bar-coded libraries were used for templating with the Life Technologies Ion PI Template OT2 Solutions 200 Kit v3 and the manufacturer’s recommended protocol. The beads prepared for sequencing were loaded onto a pre-prepared and calibrated Ion P1 chip, as directed by the Life Technologies Ion P1 Sequencing 200 Kit v3 protocol. The chip was placed into an Ion Proton sequencer and the run commenced by using a Ion torrent miRNAseq run plan that was configured according to type of library, species, number of run flows required, type of plug-in required, adapter-trimming, as well as other parameters specific to miRNAseq runs.

After completing the proton run, raw sequences were aligned to the human Hg19 build reference sequence by the Life Technologies Ion Torrent Suite. The aligned BAM files were used for further analyses. The BAM files, separated by specific bar codes, were uploaded to the Strand NGS software (San Francisco, CA, USA). Quality control was assessed by the Strand NGS programme, which determined the pre- and post-alignment qualities of the reads per sample. The aligned reads were filtered based on an alignment score, match count, mapping quality and average base quality.

### Real-time qPCR validation of a novel miRNAs signature from the plasma of both the AIS patients and healthy controls

Reverse transcription reactions were performed using the TaqMan miRNA Reverse Transcription kit, miRNA-specific stem-loop primers (Part No. 4366597, Applied Biosystems, Inc) and 100 ng of input cell-free RNA in 15 µL of the RT reaction. Real-time PCR reactions were performed in triplicate in scaled-down 10-µL reaction volumes using 5 µL of TaqMan 2 × Universal PCR Master Mix with No UNG, 0.5 µL of TaqMan Small RNA assay (20 × ) (Tables 2), 3.5 µL of nuclease-free water and 1 µL of the RT product. Real-time PCR was carried out in an Applied BioSystems 7900HT thermocycler (Applied Biosystems Inc, Foster City, CA, USA), programmed as follows: 50 °C for 2 min, 95 °C for 10 min, followed by 45 cycles of 95 °C for 15 s and 60 °C for 1 min. Raw data were analysed with version 1.0.3 of the Expression Suit Software (Life Technologies).

### Prediction of miRNA targets and over-representation analysis

A large number of potential target sites exists for any given miRNA. The computational approach to predict the targets of miRNAs facilitates the process of narrowing down all these targets. In our approach we first used DIANA-microT-CDS, accessed from the DIANA web server v5.0^67^. This tool shows if the target was also predicted by miRanda or TargetScan, or was experimentally validated in TarBase v7.0. We used the DIANA-miRPath v3.0 functional analysis online, which is suitable for identifying the miRNAs that control significant molecular pathways annotated on the Kyoto Encyclopedia of Genes and Genomes (KEGG) by using the following as the default parameters: experimentally supported interactions from DIANA TarBase v.7.0, a p-value threshold of 0.001, and a microT threshold of 0.8. To reduce the number of false-positive miRNA targets, we applied a false discovery rate (FDR) correction to select the KEGG pathways. The algorithm utilised in this analysis consisted in a one-tailed Fisher’s exact test^68^.

### Statistical modelling

Bioinformatics and biostatistics analyses were performed in the Biostatistics unit at the Instituto de Investigación Sanitaria IISLaFe (Valencia, Spain). Firstly, Deseq normalisation was performed on the raw counts data^69^ by estimating size factors using the geometric means of the transcript counts. After normalisation, variance stabilising transformation (VST) was applied prior to modelling. We used a random forest algorithm^70^ as a classifier for our model, and model performance was assessed using 20 repetitions of 10-fold cross-validations. Biomarker selection was based on the variable importance reported by the random forest algorithm using a cut-off value of 0.15 based on a screen plot with all the variables placed in order from more to less important values. A differential expression analysis was also performed based on the negative binomial distribution using the Robinson and Smyth exact negative binomial test^71^ and applying false discovery rate correction to the obtained p-values. Prior to performing the tests, samples were adjusted to an equal effective library size by thinning raw counts. To analyse the qPCR validation sample, a standard logistic regression model was performed, including our previously selected miRNA signature as predictors. Model performance was assessed by estimating a ROC curve and computing the AUC. Since the proportion of cases and controls in our sample was not representative of that in the population, we corrected the bias in the intercept of our logistic regression model with the true prevalence of the cases in our population, 4%, according to the procedure of Hosmer and Lemeshow^72^.

All the statistical analyses were performed with the R software (version 3.1.2) and DESeq. 2 (version 1.6.3)^73^, MLSeq (version 1.2.0) and NBPSeq (version 0.3.0) R-packages.

## Electronic supplementary material

Supplementary information
