# Supplementary material for: Circulating miRNAs as diagnostic biomarkers for adolescent idiopathic scoliosis
Source: Sci Rep. 2018 Feb 8;8:2646. doi: 10.1038/s41598-018-21146-x (PMC5805715; doi:10.1038/s41598-018-21146-x)
Supplement: Supplementary file 1 — Supplementary information [file 41598_2018_21146_MOESM1_ESM.pdf]

Supplementary Information

Title: Circulating miRNAs as diagnostic biomarkers for adolescent idiopathic scoliosis

Authors: José Luis García-Giménez[1,2,3]*, Pedro Antonio Rubio-Belmar[4,5], Lorena Peiró-Chova[2], David Hervás[6], Daymé González Rodríguez[2], José Santiago Ibañez-Cabellos[2,3], Paloma Bas Hermida[4,5], Salvador Mena-Mollá,[3] Eva María García-López,[2,3] Federico Vicente Pallardó Calatayud[1,2,3], Teresa Bas[4,5]

Supplementary Information. Radiological and clinical evaluation of AIS patients

It was mandatory to include from skull to pelvis. Risser method was used for skeletal maturity assessment while the Cobb method was used to measure the coronal deformity. On the sagittal plane, T5 -T12 kyphosis (normal values were assigned from 10º to 40º), T12-S1 lordosis (normal values were assigned from 37º to 47º), pelvic incidence (normal values 47º-57º) and pelvic tilt (normal values 9º-15º) were measured. Finally, coronal (C7-CSVL lines) and sagittal (C7-S1 lines) balance were taken into account. Classification of the deformity for each patient using The Lenke Classification System for Scoliosis was also collected. For control group, complete anamnesis to ensure that the patient doesn´t have any of the exclusion criteria, anthropometric variables [Age (years), high (cm), weight (Kg), BMI (kg/cm2)], complete physical and neurological examination, Adams' test, pelvic and shoulder balance were performed. Scoliometer test was applied to ensure there is no rotation in control subjects

Supplementary Information. Radiological and clinical description of AIS patients

From a clinical perspective of the evaluation of AIS patients, the mean TRACE punctuation was 6.60 (±1.66). The mean coronal plumb test value was 1.18 cm (±1.15). Using a scoliometer, on Adams Test, the mean prominence measured was 6.76º for thoracic trunk and 4.41º for the lumbar segment. Neurological exploration was normal in all patients.

Radiographic studies evidenced on the sagittal plane a T5-T12 mean kyphosis of 23.06º (±11.90) and T12-S1 mean lordosis of 56.46º (±11.10), while pelvic values were 46.77º as mean pelvic incidence (±9.48) and 11,2º as mean pelvic tilt (±5.38) (Supplemental Table 2). The mean value for radiologic coronal imbalance on the patients group was 1.05 cm (± 1.23), while the sagittal imbalance measured on X-ray was -0.15 cm (±0.25). Following Lenke classification, curve measurements were divided into proximal thoracic (PT), main thoracic (MT) and thoraco-lumbar/lumbar (TL/L); the mean Cobb´s angle on AIS group were 30º for PT curves, 34,03º

(±16,93) for MT curves and 31,31º (±11,06) for TL/L curves. The evaluation of scoliosis by specific and general health questionnaires showed that the mean punctuation for SRS-22 was 4.12 points (±0.47), 39.64 points on CAVIDRA test (±12.08) and finally a mean result of 83.56 points (±14.07) on SF-36 form. According to radiologic results, the mean skeletal maturity by Risser method was 3.46 (±1.10).

Physic exploration showed mean coronal plumb test values of 0.26 cm (±0.66), thoracic prominence of 0.7º (±1.49) while the mean lumbar hump value was 1º (±1.41). A normal neurologic exploration was established in all individuals (Table 1).

According to X-ray results, the Risser evaluation was 2.46 (±1.80). On anteroposterior view the mean Cobb angle measured was 2.23º (±3.37º). Sagittal radiologic analysis showed a mean T5-T12 kyphosis of 34.92º (±10.16) and a mean T12-S1 lordosis of 63,46º (±11.34). No coronal or sagittal imbalance was detected clinically or radiologically. Pelvic parameters were this time a mean pelvic incidence of 43.92º (±5.89) and a mean pelvic tilt of 8.8º (±3.40). SF-36 form resulted on a mean punctuation of 85.93 points (±8.04) (Table 1).

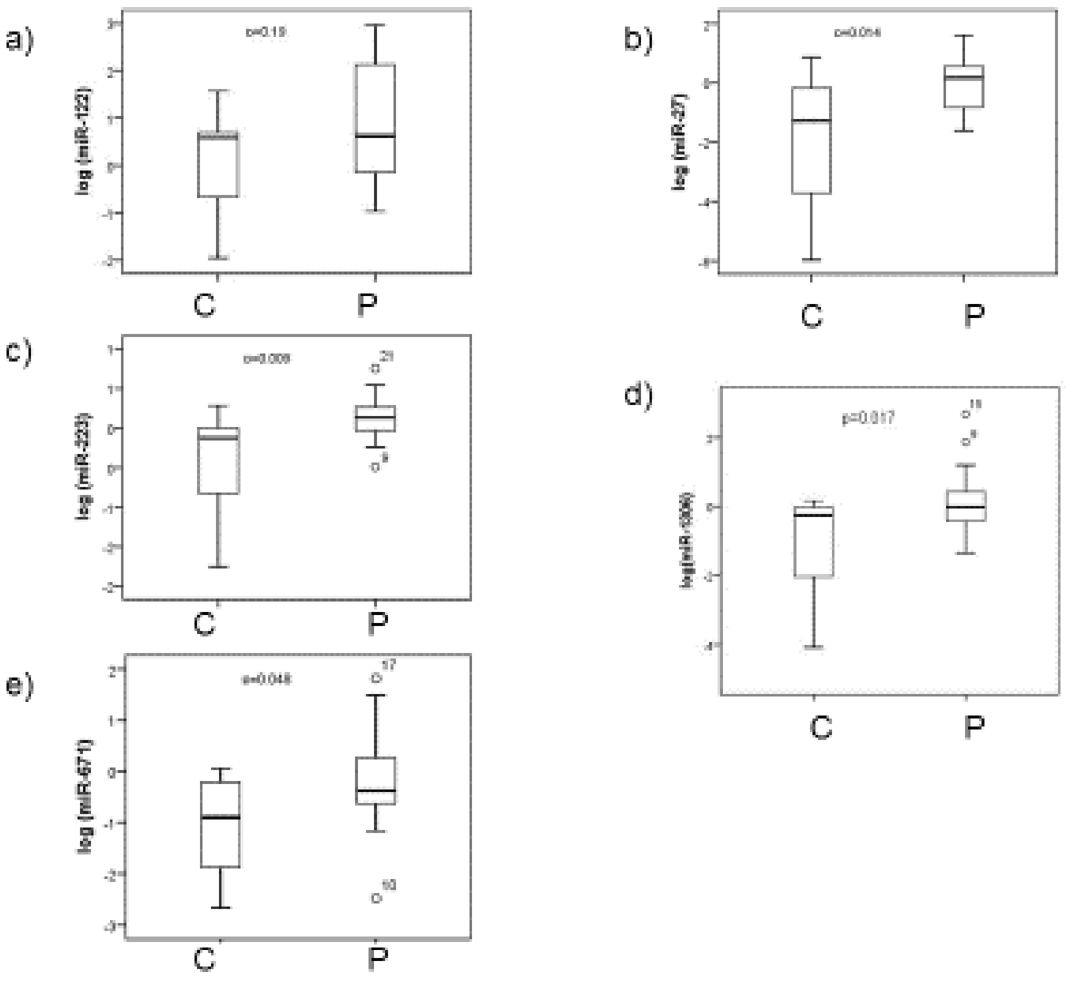

Figure S1. Relative expression levels of the miRNAs with different representation found in plasma of patients of AIS compared to control healthy subjects in the validation cohort. Box plot of relative expression levels of miRNAs analyzed by RT-qPCR normalized to miR-191 as endogenous control and calculated using the $2^{-\Delta\Delta Ct}$ method. a) miR-122 (Fold Change, FC > 1.70; p > 0.05; b) miR-27a (FC >3.90; p < 0.05); c) miR-223 (FC > 1,51; p < 0.05); d) miR-1306 (FC > 2,58; p < 0.05); and e) miR-671 (FC > 2.42; p < 0.05). Samples have been ordered according to their corresponding group controls (C) or AIS patients (P). An independent samples t-test was applied for analyzing biospecimens from 7 healthy subjects (3 male and 4 female) and 17 AIS patients (2 male and 15 female). p < 0.05 was considered to indicate a significant difference.
